# Versatile roles of polyamines in improving abiotic stress tolerance of plants

**DOI:** 10.3389/fpls.2022.1003155

**Published:** 2022-10-13

**Authors:** Jinhua Shao, Kai Huang, Maria Batool, Fahad Idrees, Rabail Afzal, Muhammad Haroon, Hamza Armghan Noushahi, Weixiong Wu, Qiliang Hu, Xingda Lu, Guoqin Huang, Muhammad Aamer, Muhammad Umair Hassan, Ayman El Sabagh

**Affiliations:** ^1^ Research Center on Ecological Sciences, Jiangxi Agricultural University, Nanchang, China; ^2^ China Guangxi Hydraulic Research Institute, Nanning, China; ^3^ Key Laboratory of Water Engineering Materials and Structures, Nanning, China; ^4^ College of Plant Science and Technology, Huazhong Agricultural University, Wuhan, China; ^5^ Department of Field Crops, Faculty of Agriculture, Siirt University, Siirt, Turkey; ^6^ Department of Agronomy, Faculty of Agriculture, University of Kafrelsheikh, Kafr El Sheikh, Egypt

**Keywords:** polyamines, abiotic stress, antioxidant system, hormonal regulation, stress tolerance

## Abstract

In recent years, extreme environmental cues such as abiotic stresses, including frequent droughts with irregular precipitation, salinity, metal contamination, and temperature fluctuations, have been escalating the damage to plants’ optimal productivity worldwide. Therefore, yield maintenance under extreme events needs improvement in multiple mechanisms that can minimize the influence of abiotic stresses. Polyamines (PAs) are pivotally necessary for a defensive purpose under adverse abiotic conditions, but their molecular interplay in this remains speculative. The PAs’ accretion is one of the most notable metabolic responses of plants under stress challenges. Recent studies reported the beneficial roles of PAs in plant development, including metabolic and physiological processes, unveiling their potential for inducing tolerance against adverse conditions. This review presents an overview of research about the most illustrious and remarkable achievements in strengthening plant tolerance to drought, salt, and temperature stresses by the exogenous application of PAs. The knowledge of underlying processes associated with stress tolerance and PA signaling pathways was also summarized, focusing on up-to-date evidence regarding the metabolic and physiological role of PAs with exogenous applications that protect plants under unfavorable climatic conditions. Conclusively, the literature proposes that PAs impart an imperative role in abiotic stress tolerance in plants. This implies potentially important feedback on PAs and plants’ stress tolerance under unfavorable cues.

## Introduction

Climate change is exacerbatingthe stress challenges for plants that are often non-conducive or stressful for plant development. Crop losses due to abiotic stresses are rising steadily and exert severe constraints on crop productivity (FAO, 2019). Moreover, plants commonly suffer from osmotic, ionic, or oxidative stresses and are projected to intensify when exposed to extreme climatic conditions, hence escalating the damage to crop productivity (Gong et al., 2020; Javeed et al., 2021). During stressful cues, plants suffer with oxidative stress by overaccumulation of reactive oxygen species (ROS) which are highly toxic and reactive radicals (peroxides, oxides, superoxide) that potentially damage the cellular assemblies and components (proteins, lipids, carbohydrates and DNA), consequently resulting in cell death (Gechev and Petrov, 2020; Kumari et al., 2021; Chattha et al., 2021). Plants respond to oxidative stress by the induction of a defensive system including enzymatic antioxidants [superoxide dismutase (SOD); catalase (CAT); peroxidase (POD); ascorbate peroxidase (APX)] and nonenzymatic antioxidants (ascorbic acid, carotenoids, phenolic content, glutathione, etc.) (Kaur et al., 2019; Hasanuzzaman et al., 2020; Zafar et al., 2020; Chauhan et al., 2022).

Polyamines (PAs) are low-molecular weight aliphatic amine compounds containing nitrogenous bases with amino groups and exhibiting a strong potential of biological activity in normal developmental processes, as well as serving indispensable functions in the tolerance of plants under unfavorable circumstances (Thomas et al., 2020; Tsaniklidis et al., 2020; Hasan et al., 2021b). Moreover, these compounds primarily exist in free, covalently conjugated (perchloric acid-soluble conjugated or -insoluble bound) or non-covalently conjugated forms in living organisms, including plants (Gill and Tuteja, 2010; Chen et al., 2019). Putrescine (Put), spermine (Spm) and spermidine (Spd) are low-molecular weight aliphatic amine natural compounds that serve vital functions in physiological and developmental processes such as cell division and proliferation, embryogenesis, leaf senescence, floral development, and responses to abiotic stress (Hasan et al., 2021b).

PAs exist as polycations at physiological pH that result in their higher electrostatic affinity for negatively charged molecules such as nucleic acids and membrane phospholipids in cells (Aktar et al., 2021), which is associated with improving enzyme activity, regulating replication and transcription processes, and modulating cell division and membrane stability, besides a wide range of biological activities related to cell development (Mustafavi et al., 2018). Soluble forms of PAs were found to be linked with small phenolic molecules (hydroxycinnamic acid, coumaric acid, caffeic acid, or ferulic acid), which give rise to a large PAs pool (serving as metabolites) in plants (Martin-Tanguy, 1997; Luo et al., 2009; Bassard et al., 2010; De Oliveira et al., 2018; Mustafavi et al., 2018). Free polyamines combine with macromolecules by covalently bonding with either ionic or hydrogen bonds (proteins, nucleic acids, uronic acids, lignin), generating insoluble bound PAs (Gill and Tuteja, 2010; Chen et al., 2019). Conjugated PAs regulate the intracellular concentration of free PAs that might be correlated with cell growth and stress tolerance (Mustafavi et al., 2018).

To overcome the severe impact of environmental stresses, the accumulation of different osmolytes (carbohydrates, betaine, proline, and other amino acids) is found to be an adaptive mechanism that plants use to maintain cellular turgor pressure and respond differently to different abiotic factors; however, alteration of primary metabolism is the most common reaction. It involves alterations in the content of various amino acids, sugars, and tricarboxylic acid cycle intermediates, exhibiting common characteristics in abiotic stress responses. Plants also undergo modification in secondary metabolite content under exposure to abiotic conditions; additionally, these alterations vary according to species and stress type (Khan et al., 2018).

It might be helpful to stress that throughout their existence, various organisms are repeatedly exposed to various stresses and develop species-specific strategies to resist them and improve survival. Drought, starvation, heat, and cold shocks are universal stressful stimuli which living creatures encounter during their life cycle. In addition, the organisms utilizing aerobic respiration or photosynthesis to produce energy continually deal with numerous oxidative challenges related to reactive oxygen and nitrogen species. Remarkably, like starvation and heat shock, oxidative stress induces polyamine synthesis in various species (Polis et al., 2021). The PA metabolic pathways are mostly conserved with slight variations from bacteria to plants and animals. In general, prokaryotes primarily synthesize Put and, to a lesser extent, Spd (Tabor and Tabor, 1976; Wortham et al., 2007), while Spm is less commonly found (Hamana and Matsuzaki, 1992; Busse, 2011; Michael, 2016). The PAs’ mode of action is broadly attributed to their cationic nature since all amino groups and most imino groups are highly protonated.

Gilad and Gilad (2003) coined the term polyamine stress response (PSR), which is a common reaction to stressful stimuli, including physical, emotional, and hormonal stressors, with a magnitude related to stress intensity. It is universally accepted that transient polyamines synthesis is induced in various species (not just in plants) by heat stress, radiation, and other traumatic stimuli in a process termed the polyamine stress response (Banerjee et al., 2021), which was observed in fish and mammals. Thus, it is ubiquitous and universal. Additionally, PAs are versatile compounds, protecting the protein structure and inducing antioxidative mechanisms, which provide tolerance against various unfavorable environmental cues (Alcázar et al., 2020). PAs are linked with plant development, stress tolerance, protection of nucleic acids and the cellular membrane and, ultimately, plant growth under stress (Sequera-Mutiozabal et al., 2016; Pál et al., 2018b). Osmolyte accumulation plays an important role in scavenging ROS to counteract the adverse effects (Alhaithloul et al., 2020). Plants have developed a well-organized mechanism that protects the cell from environmental adversities and amino acids; metabolism is one of these mechanisms. Nonetheless, exogenously applied PAs led to higher antioxidant enzyme (SOD, POD, CAT) activities under stress (Nahar et al., 2016; Sánchez-Rodríguez et al., 2016a; Hassan et al., 2018). Besides the direct safeguard, PAs as signaling molecules regulate several vital metabolic mechanisms. Pál et al. (2015) documented that the PA’s functions progressively act in stress signaling rather than in PA’s accumulation that substantially enhances abiotic stress adaptations. PAs effectively remove excessive ROS, in turn reducing cell damage, thus enhancing stress tolerance. In this review, we deepen the knowledge about the important links between PAs and events under abiotic stress that imparts successful defense in additional crops. The interplay between osmotic substances and PAs are discussed as well.

## Biosynthesis of polyamines

**Figure 1:**
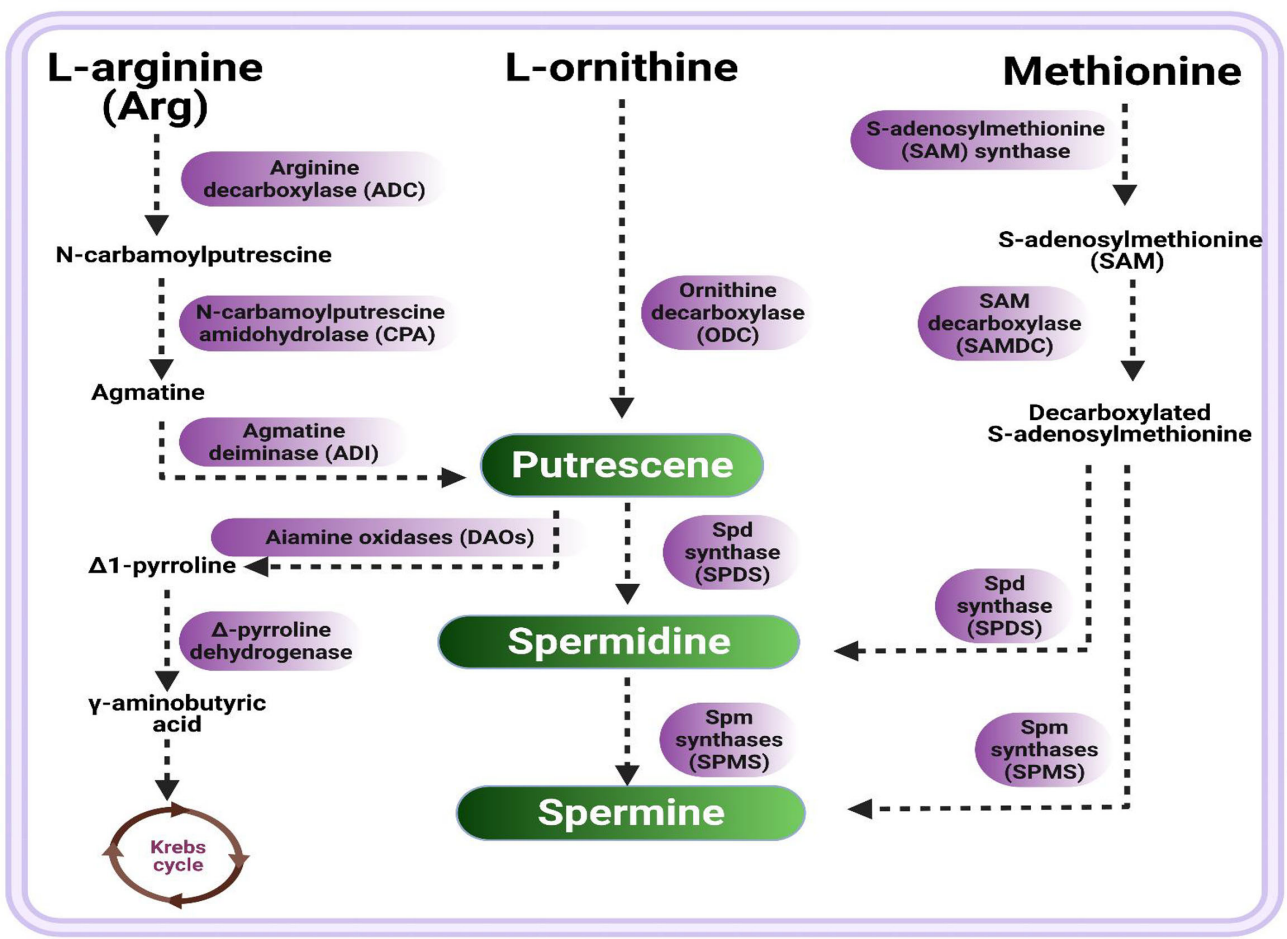
Schematic presentation of polyamines biosynthetic pathway. There are three routes of putrescine synthesis from arginine (route 1), ornithine (route 2), and methionine (route 3) followed by multiple sequential steps, resultant putrescine converts into spermidine which in turn produce spermine.

PAs are aliphatic amine compounds comprising variable hydrocarbon chains and amino groups (Sequera-Mutiozabal et al., 2017). Previous studies revealed that the metabolism of PAs could be well-represented in PA biosynthesis pathways, including various anabolic and catabolic processes (Alcázar et al., 2010; Sequera-Mutiozabal et al., 2017; Handa et al., 2018). A diagrammatic presentation of PAs’ biosynthesis-related metabolic pathways is shown in **Figure 1**. In plants, diamine putriscene is derived either through multiple sequential process of L-arginine decarboxylation catalyzed by arginine decarboxylase (ADC) followed by two successive steps catalyzed by agmatine iminohydrolase (AIH) and N-carbamoylputrescine amidohydrolase (CPA), or by L-ornithine through catalysis of ornithine decarboxylase (ODC). Under unfavorable cues, PAs’ synthesis is induced by the arginine decarboxylase (ADC) pathway (Slocum et al., 1984; Bouchereau et al., 1999; Bano et al., 2020). Moreover, arginine is first converted into N-carbamoylputrescine by a reaction catalyzed by arginine decarboxylase (ADC), and decarboxylated arginine (agmatine) is synthesized by plants (Coleman et al., 2004; Pegg, 2016), then agmatine is converted into diamine putrescine (Put) via an intermediate N-carbamoylputrescine by N-carbamoylputrescine amidohydrolase (CPA), followed by agmatine deiminase (ADI)-catalyzed reactions. The diamine putrescine works as a precursor that leads to the production of Spd and Spm via successive attachment of the aminopropyl group. First, Put produced triamine Spd through Spd synthase (SPDS) catalysis; in turn, Spd will produce tetramine spermine (Spm) by Spm synthases (SPMS). Spd is also converted to an Spm isomer, thermospermine (T-Spm); a reaction catalyzed by T-Spm synthase. Spm and Spd serve as substrates for synthesis of PAs (Knott et al., 2007). Additionally, putrescine is subjected to catabolism by diamine oxidases (DAOs), converted to Δ1-pyrroline with the release of byproducts; i.e., ammonia and H_2_O_2_, followed by Δ1-pyrroline degradation into γ-aminobutyric acid by Δ1-pyrroline dehydrogenase, which is ultimately changed into succinic acid (Krebs cycle component) (Eller et al., 2006). The biosynthesis of Spd takes place through S-adenosylmethionine (SAM) decarboxylation by SAM decarboxylase (SAMDC), in which an aminopropyl group works as a substrate which is added with Put to generate Spd, followed by the addition of another aminopropyl moiety (derived from decarboxylated SAM) catalyzed by Spm synthase to form Spm (Slocum et al., 1984) (**Figure 1**). Furthermore, free PAs’ contents are maintained during normal as well as unfavorable circumstances by PA catabolic pathways.

**Figure 2:**
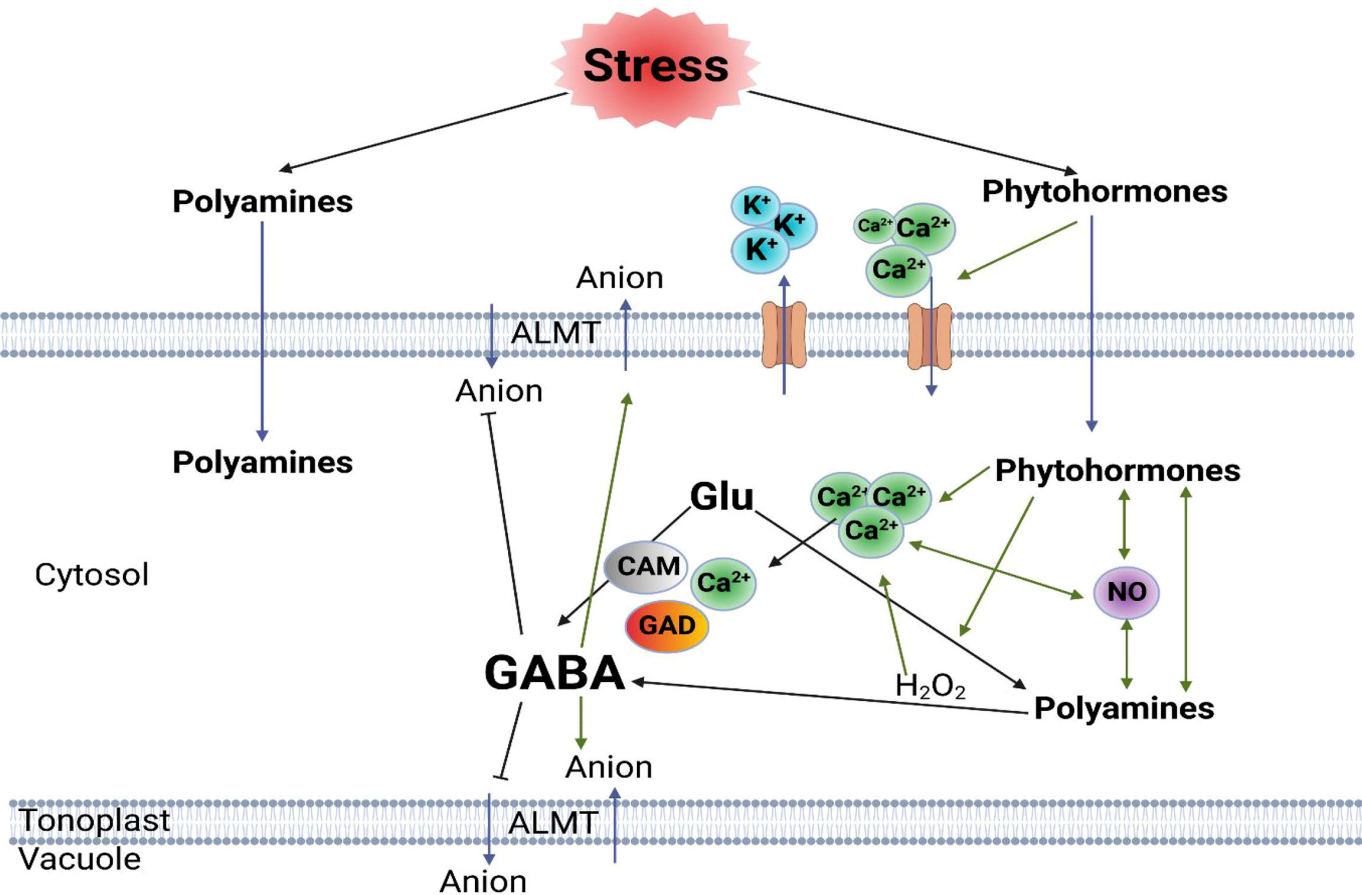
Diagrammatic model of plant stress responses mediated by hormones, polyamines and GABA in different plant cell types, including guard cells and root cells based on Bown and Shelp (2016). Continuous black arrows indicate metabolic pathways; green arrows indicate regulation process; and blue arrows indicate transportation. Glu; L-glutamate, GABA; γ-aminobutyric acid, ALMT; aluminium-activated malate transporter, CAM; calmodulin, GAD; glutamate decarboxylase. (Marco et al., 2011; Choi et al., 2016; Podlešáková et al., 2019).

In addition, Arg might play a crucial role in stress recovery, as it is the most versatile amino acid linked to the biosynthesis of signaling molecules. Arginine has an important role in nitrogen metabolism in germinating seeds and in developing seedlings (Hasanuzzaman et al., 2018). The crucial roles of Arg and NO in enhancing drought stress tolerance in wheat seedlings by upgrading their water status and reducing oxidative stress and MG toxicity. Thus, it not only serves as a protein constituent but is also a precursor of polyamines, agmatine, proline, and the cell signaling molecules glutamine and nitric oxide (Liu et al., 2006; Hasanuzzaman et al., 2018). Further, exogenous application of arginine increased the antioxidant activity, total content of phenolic compounds, polyamines, and proteins under heat stress (Collado-González et al., 2020). Previous reports documented a significant connection between PAs and hormones regulating plant defense systems and described that plants overexpressing arginine decarboxylase (ADC2) revealed both up- and down-regulation of hormone-related genes and encoding transcription, genes involved in the biosynthesis of auxin, ethylene (ET), abscisic acid (ABA), jasmonates (JA), and salicylic acid (SA); genes responsible for auxin transport, auxin-responsive proteins, ET- and ABA-responsive transcription factors, and JA-induced proteins (Marco et al., 2011). They also showed that these plants had alterations in Ca^2+^ signaling. Additionally, it was indicated that long-distance signaling by these hormones is mediated by waves of ROS and Ca^2+^ rather than directly by the movement of the hormones themselves (Choi et al., 2016), which concluded that the stress response is a complex interaction between all plant hormones, PAs’ and ROS response, NO production and the levels of Ca^2+^ (Marco et al., 2011; Choi et al., 2016; Podlešáková et al., 2019), illustrated in **Figure 2**. Taken together, PAs are endogenous plant growth regulators or intracellular messengers that modulate various complex physiological processes such as abiotic stress responses, indicating that PAs are important for cell survival (Chen et al., 2019).

## Polyamines and abiotic stresses

### Polyamines and cold stress

Low temperature at an early stage leads to a reduction in germination and unwell seedling establishment; in addition, at the reproductive phase, this leads to delays in heading and causes pollen sterility, resulting in a decrease in grain yield (Anwar et al., 2021). Several studies have been documented that exogenous PAs’ application can enhance low-temperature tolerance (Todorova et al., 2015). Likewise, priming of putrescine (Put) increased seed germination and seedling development besides enhancing tolerance for low-temperature stress, compared with non-primed fennel seeds (Mustafavi et al., 2015). *Anthurium andraeanum* under chilling stress, subjected to the exogenous application of Put, showed lower membrane injury and malondialdehyde (MDA) content, enhanced antioxidant and proline levels, evidencing that the application of exogenous Put could effectively reduce damage (Sun et al., 2020). A previous report identified 1273 differentially expressed gene (DEG) that were characterized into three groups: biological process, molecular function, and cellular component; the identified DEG in the biological process group were related to defense responses and responses to abscisic acid, oxidative stress, and water deficit; the molecular function group was assigned with DEG related to ions and ATP-binding processes that promote the binding capacity of ions and ATP; the cellular component group includes DEGs involved with cellular components such as cytoplasm, nucleus, and plasma membrane, suggesting the participation of Put in the maintenance of membrane integrity (Sun et al., 2020). Additionally, Put elevated the tolerance by alleviating H_2_O_2_ and MDA levels via modulating the antioxidant system, besides enhancing the level of free and conjugated PAs in tomatoes under chilling stress (Song et al., 2014). Furthermore, Put and Spd treatment increased the expression level of the 9-cis-epoxycarotenoid dioxygenase (LeNCED1), and enhanced nitric oxide (NO) content by H2O2 dependent signaling via the NR and NOS-like pathways and hence, enhanced the level of Put which induced tolerance (Diao et al., 2017), coinciding with earlier reports obtained in *Arabidopsis* (Cuevas et al., 2009). Moreover, PAs’ supplementation increased the accretion of osmotic substances, including proline, carbohydrates, and glycine betaine, resulting in abiotic stress (low temperature) tolerance in *Stevia rebaudiana* plants (Moradi Peynevandi et al., 2018). The Put inoculation on peach quality under low temperature during storage indicated that Put significantly slowed the fruit softening rate, fading of fruit skin color, and reduction of fruit weight, ascorbic acid content, total soluble solids and titratable acidity irrespective of Put doses and time of application (Abbasi et al., 2019).

Spermidine (Spd) treatment on cucumber plants before exposure to chilling stress revealed that plants had improved chlorophyll level and growth (He et al., 2002). Furthermore, Spd pre-treatment lessened the decrement of chlorophyll fluorescence yield and photosynthetic efficiency and reduced the damage of thylakoid membranes, consequently raising chilling tolerance with the protection of the photosynthetic apparatus in cucumber (He et al., 2002). Regarding spermidine synthase (SPDS), cDNA from *Cucurbita ficifolia* was introduced into *Arabidopsis* (Kasukabe et al., 2004); moreover, the transgenic plants showed a significantly higher SPDS activity and Spd level in leaf tissues which improved tolerance against low-temperature stress (Groppa and Benavides, 2008).

Spd pre-treated mung bean seedlings reduced low-temperature injury through the modulation of the ascorbate–glutathione pathway and reduction of the components in the glyoxylate cycle, which decreased the oxidative stress (Nahar et al., 2015). Spd seed priming in rice increased the alpha-amylase activity and osmolytes and improved antioxidant capacity, resulting in improved tolerance to chilling stress (Sheteiwy et al., 2017). In addition, Kou et al. (2018) demonstrated that the arginine decarboxylase gene (*ADC1*), associated with the putrescine pathway, plays a vital role in cold-acclimated potato freezing tolerance. Moreover, Wang et al. (2011) have shown that an arginine decarboxylase gene (*PtADC*) from *Poncirus trifoliata* confers abiotic stress tolerance and promotes primary root growth in *Arabidopsis*.

Exogenously applied Put or Spd enhanced the activity of enzymatic antioxidants, thereby, indicating the protective role of PAs in chilling-tolerant centipedegrass (Chen et al., 2018). It is reported that abiotic stress tolerance is mainly due to PAs’ functions during signal transduction instead of PAs’ accumulation (Pál et al., 2015). The exogenously applied PAs (Put, Spd and Spm) improved the proline contents, regulated the functioning of H^+^-ATPase of plasma membrane, and delayed the stimulation of ethylene emission under cold stress, suggesting that PAs might act as elicitors that stimulate the defensive response, which leads to compensating the adverse effect of low-temperature stress in winter oilseed rape (Jankovska-Bortkevič et al., 2020). The previous findings on transgenic plants revealed that the biosynthesis of PAs was attributed to higher stress tolerance (Kusano et al., 2008). The Spm application maintained higher endogenous Spm and Spd levels, hindering Put accumulation, thereby decreasing chilling stress (Roy and Wu, 2001).

### Polyamines and heat stress

The exogenous PAs’ application that influenced plant tolerance to high temperature (heat stress) has been reported in several studies (Chen et al., 2019). Exogenous PAs’ application might ameliorate high-temperature stress by maintaining membrane integrity and inducing antioxidant defense systems (Kumar and Mallick, 2018). Previous study demonstrated the effects of heat stress on the accumulation of proline and the level of PAs in tobacco plants (Cvikrová et al., 2012). An earlier study documented that PAs interacted with melatonin and enhanced the heat tolerance of plants, which was evidenced by melatonin exogenous application that positively improved heat tolerance in tomatoes via enhancing the antioxidant system efficiency, activating the ascorbate–glutathione cycle, and reprogramming the PAs’ metabolic and NO biosynthesis pathways that aid in ROS detoxification and improved cellular membrane stability to lessen heat-induced oxidative stress (Jahan et al., 2019). Putrescine (Put) is an initial product in the PA biosynthesis pathway; reports have elaborated that it displays its function in tolerance of oxidative stress through hormonal regulation and stress signaling in plants (Pál et al., 2018a); moreover, exogenous application of Put had improved heat stress tolerance in tea plants, where it improves pollen performance and reduces ROS which is dose-dependent (Çetinbaş-Genç, 2020). In tomato seedlings, exogenous Put improved heat tolerance by improving chlorophyll content and reducing chlorophyll catabolic enzyme activity, regulating endogenous free polyamines, elevating antioxidant defense capacity, and inducing heat-shock-related gene expression (Jahan et al., 2022).

In soybeans, the effect of Put, Spd, and Spm on heat-shock protection showed improvement in root and hypocotyl development besides lower electrolyte leakage and MDA content in various tissues, indicating membrane integrity that might be due to PAs’ replacement with Ca^2+^ by binding to membrane phospholipids (Amooaghaie and Moghym, 2011). In wheat plants, Put application before high-temperature treatment results in higher tolerance by higher PA and amino acid levels by decreasing the toxic product (ethylene and NH^4^) (Hassanein et al., 2013). Genome-wide expression profiles of tomato fruits under high-temperature stress with exogenous Spd were studied and showed that Spd application regulated various signal transduction factors and PA biosynthetic and hormone pathway genes, where the major proportion of regulated genes (361 genes) includes 49 loci (13.57%) that were related to stress signaling, along with a group of transcription regulation and sugar metabolism-related genes, plus a group of upregulated genes related to hormonal signaling that subsequently play an important role in regulating tomato fruit response to high temperatures during the ripening stage (Cheng et al., 2012).

Proteomic approaches were used to inspect the impact of exogenous Spd, which showed that most identified proteins were related to photosynthesis, inferring photosynthetic efficiency in tomato seedlings under high-temperature stress (Sang et al., 2017). Furthermore, the exogenous Spd might be related to the higher expression level of proteins contributing to the cellular defense and antioxidant enzyme system-related gene expression during higher heat stress in tomato seedlings (Sang et al., 2017). Application of Spd and Spm maintained the plant water status and enhanced the chlorophyll content, antenna conversion efficiency, stomatal conductivity, transpiration, quantum yield of photosystem II and photochemical quenching coefficient of flag leaves in spring wheat under high-temperature stress (Jing et al., 2019).

**Figure 3:**
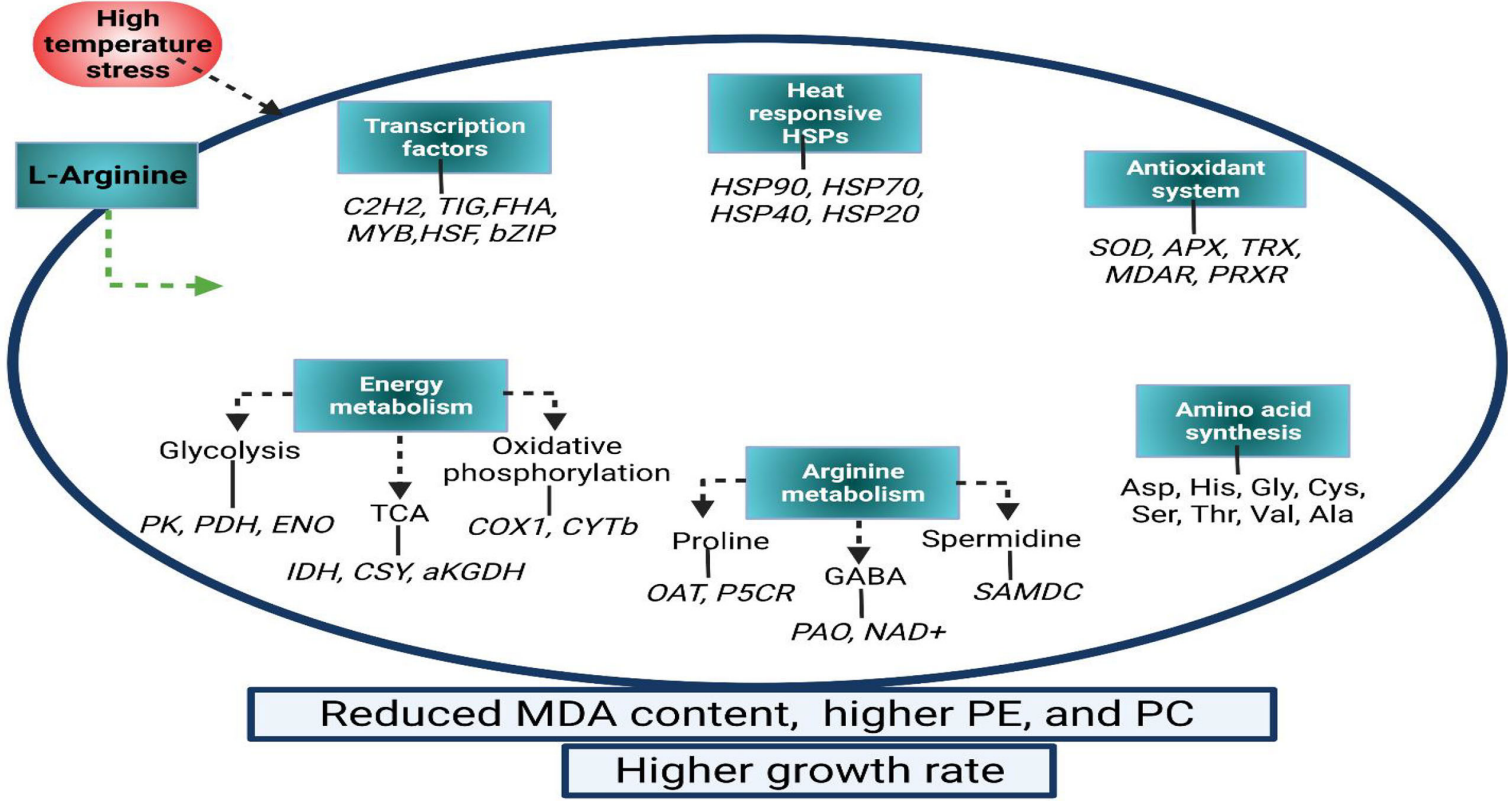
A model diagram for Arg-induced high temperature tolerance in *G. lemaneiformis* attributed to physiological and transcriptional alterations. PE, phycoerythrin; PC, phycocyanin; MDA, malondialdehyde; SOD, superoxide dismutase; APX, ascorbate peroxidase; TRX, thioredoxin; MDAR, monodehydroascorbate reductase; PRXR, peroxiredoxins; OAT, ornithine–oxo-acid transaminase; P5CR, pyrroline-5-carboxylate reductase; SAMDC, S-adenosylmethionine decarboxylase; GABA, γ-aminobutyric acid; PAO, polyamine oxidases; NAD+, aldehyde dehydrogenase; Asp, aspartate; His, histidine; Gly, glycine; Cys, cystine; Ser, serine; Thr, threonine; Val, valine; Ala, Alanine; PK, pyruvate kinase; PDH, pyruvate dehydrogenase; ENO, enolase; TCA, tricarboxylic acid; IDH, isocitrate dehydrogenase; CSY, citrate synthase; αKGDH, 2-oxoglutarate dehydrogenase (Zhang et al., 2021).

Polyamines (Spd and Spm) and POD functioning are positively related to grain weight in two wheat varieties with different tolerance capacities under heat stress, where enzymatic antioxidant activity and the content of Spm, Spd and proline (Pro) were remarkably elevated via Spd/Spm exogenous application (Jing et al., 2020). Moreover, in trifoliate orange plants, the exogenous Spm treatment may increase the tolerance to combined stress by enhancing antioxidant enzyme activities and ROS quenching which prevent cellular injury and membrane damage under combined (high temperature and drought) stress conditions (Fu et al., 2014). Likewise, pretreatment of Spm in mung beans improved the tolerance against high temperature, drought, and combined stresses (Nahar et al., 2017). Exogenously applied arginine significantly improves the tolerance against abiotic stress damages in various crops (Nasibi et al., 2013; Karpets et al., 2018; Silveira et al., 2021). Similarly, arginine is significant in combating a non-conducive environment through arginine-derived stress-related substances under high-temperature stress in *G. lemaneiformis* (Zhang et al., 2021) (**Figure 3**).

### Polyamines and water stress

Drought is a serious challenge in agricultural production due to its disruptive effects on key physiochemical processes in plants (Hassan et al., 2021; Zahoor et al., 2021). The interplay of PAs and water stress is a very important aspect to study in the context of expanding global warming conditions. Several reports documented the association of polyamines and water stress, mainly focusing on water deficit tolerance (Ebeed et al., 2017a), while few studies were presented in the context of waterlogging tolerance. PAs including Put, Spm, and Spd might be involved in regulating the potassium ion (K^+^) channels and size of guard cells’ apertures, thus significantly affecting the regulation of the opening and closing of pores and water movement (Liu et al., 2000). Various studies found that exogenous Put treatment at optimum concentration stimulated different physiological processes and osmotic substances (soluble sugars, amino acids, and proline) that lead to stress tolerance and higher product quality (Sánchez-Rodríguez et al., 2016b; Mohammadi et al., 2018). Put application under drought stress enhanced the seed germination and growth-related traits in alfalfa (Zeid and Shedeed, 2006). It was found that the Spm mutants (*acl5*/*Spms*) were highly sensitive under drought and salt stress conditions, which showed the significance of Spm in *Arabidopsis* under non-conducive conditions (Yamaguchi et al., 2007). In cherry tomatoes, PAs were found to be significantly related to drought tolerance (Montesinos-Pereira et al., 2014). Spd and Spm were strongly related to relief of inhibitory drought effects and increased the grain filling in wheat, whereas Put showed contrasting behavior (Liu et al., 2016a). It was concluded that PAs might differ according to plants and different parts within the same plants under osmotic stress (Sen et al., 2018); therefore, special attention is needed for the application of PAs for stress tolerance in plants. Under drought stress, PA application leads to higher stress tolerance in the different studied plants (Todorova et al., 2015). Put foliar application in wheat plants improved the plant water content, photosynthetic pigments, and osmotic substances (proline, amino acids, and soluble sugar content) under water stress, resulting in higher plant height, enhanced leaf area and improved grain yield (Gupta et al., 2012).

One of the important plant reactions is maintaining cellular water status under drought stress. Put pretreatment reduced water loss and retained higher photosynthetic efficacy, indicating that Put modulated plant tolerance against osmotic stress (Kotakis et al., 2014). Furthermore, Spd treatment enhanced the level of GA and improved the defense system by elevating antioxidative enzymatic activity and transcription mechanism, particularly ascorbic acid (AsA); additionally, various metabolites, including total phenols, flavonoids, proline, metallothionein, and cysteine content have been enhanced, reducing the destruction of photosynthetic apparatus under drought stress in white clover (Li et al., 2016). Moreover, recent reports have documented that PAs improved tolerance to water deficit conditions by elevating endogenous PA content in wheat and maize (Ebeed et al., 2017b; Kutlu Çalişkan et al., 2017).

In lettuce, foliar application of Put results in lower stomatal density, sustained ultrastructure of chloroplast and lower plasmolysis that improves water use efficiency, resulting in drought tolerance (Zhu et al., 2019). Moreover, Spd pretreatment improved leaf growth under drought and salt stress in bermudagrass plants; comparative proteomic analysis displayed that proteins involved in electron transport and energy pathways were enriched, in addition to nucleoside diphosphate kinase (NDPK) and antioxidant enzymes being regulated under polyamine application in *Cynodon dactylon* (Shi et al., 2013). Application of Spd or Spm conferred dehydration resistance by improving enzyme activities, proline content, and photosynthetic pigments against drought stress (Mustafavi et al., 2016). Spm pretreatment improved drought tolerance through modulating antioxidative capacity and stomatal closure (Shi et al., 2010). Foliar application of Spd in finger millet (*Eleusine coracan*a L. Gaertn.) plants displayed a defense to counter chlorophyll degradation and lower electrolytic leakage and hydrogen peroxide (H_2_O_2_), as well as proline accumulation, to alleviate the drought stress (Satish et al., 2018).

Spm or Spd supplementation enhances the water status of plants, stimulates antioxidant activity, alters the PAs’ metabolism, and improves water stress tolerance in Damask rose (Hassan et al., 2018). Early seedling growth is highly susceptible to drought events; moreover, water deficiency reduced the seed germination besides delayed germination and seedling establishment (Yan, 2015). Thus, it is imperative to launch appropriate tactics to mitigate the adverse impact on germination under drought stress. Seed priming is one of the pretreatments in which the seeds are exposed to a particular solution that permits partial hydration without radicle protrusion (El-Badri et al., 2021). Priming assists in initiating several physiological processes related to early seed germination; however, it averts the transition to the germination process (Ibrahim, 2016). Various techniques, including hydropriming, halopriming, osmopriming, and chemical priming are frequently used (Savvides et al., 2016). After priming, seeds are removed from the solution and are re-dried to maintain the advantageous impacts of the priming application without losing the quality (Ibrahim, 2016). After primed seeds sowing, the embryos’ puffiness in seeds accelerate seed germination by improving water uptake. It facilitates the initiation of pre-germination metabolic processes, that cause seedlings’ rapid emergence, vigorous growth, and better performance under severe and non-conducive environments; thus, shielding the seeds from unfavorable cues during the sensitive steps of seedling establishment (Ibrahim, 2016; El-Badri et al., 2021). Chemical priming is a promising technique that helps seed germination under stress conditions through improving physiological attributes; hence, stress management (Savvides et al., 2016).

Polyamines are one of the groups of chemicals that work as priming agents and might confer improved abiotic stress tolerance (Savvides et al., 2016; Tiburcio and Alcázar, 2018). In maize, Put priming was useful in enhancing the germination process and seedling establishment, improving growth under dehydration conditions (Hussain et al., 2013). Further, Spd priming imparts positive effects on seed germination upon dehydration in white clover seeds, where it accelerates seed germination and seedlings’ vigor by root elongation and improved weights (Li et al., 2014). It was premised that Spd priming application improves stress tolerance by enhancing amylase activities (Li et al., 2014). Moreover, Spd and Spm elevated the hormonal status, starch degradation, and soluble sugar level under water deficit conditions, which improved the germination of wheat plants subjected to stress (Liu et al., 2016b).

PAs (particularly Put and Spm) priming enhanced the osmoprotectants and regulated the PA biosynthesis gene expression in wheat (Ebeed et al., 2017b). Additionally, seed treatment with PAs (combined Put + Spd + Spm) led to the accumulation of osmotic substances, reducing oxidative stress and maintaining water status in mung bean in water deficit conditions (Sadeghipour, 2019). PA application in wheat exposed to drought stress resulted in better growth and photosynthetic capacity, increased levels of proteins and Rubisco, as well as improved chloroplast structure (Hassan et al., 2020). Additionally, PA foliar application showed a marked alleviation of electrolyte leakage, MDA level, and Na^+^/K^+^ ratio, in addition to higher catalase (CAT) activity and reduced ROS overgeneration (Hassan et al., 2020). The important roles of exogenous PAs applications through inducing various morpho-physiological modifications to provide stress tolerance are shown in **Table 1**.

**Table 1:** Exogenous PAs application triggered desiccation resistance in different plant species.

Crop type	Polyamine treatments	Ways of action and outcomes	Citations
*Triticum aestivum* L.	PAs (Spd and Spm)	Counteracted the negative influence of water deficit conditions at grain-filling stage.	Liu et al., 2016a
*Agrostis stolonifera*	PAs (Spd and Spm)	Improved the product quality, hydration status, photochemical efficacy, membrane integration, and hormonal regulation (GA1, GA4, and ABA); consequently, higher stress tolerance.	Krishnan and Merewitz, 2017
*Thymus vulgaris* L.	Putrescine (Put) application	Maintained the water potential of leaves, dry matter conservation, and strong antioxidant system that led to stress tolerance.	Mohammadi et al., 2018
*Triticum aestivum* L.	Proline (Pro) application	Ameliorated the dehydration stress at tillering and anesthesia stages; hence, improved the yield.	Gupta and Thind, 2017
*Chenopodium Qunioa*	Foliar spray of GB	Improved growth parameters, relative water content, photosynthetic pigments, indoleacetic acid, phenolics, TSS, Proline, and free amino acids in leaf tissues.	Elewa et al., 2017
*Citrus reticulata* Blanco	Spm application	Improved plant defense system for dehydration tolerance.	Shi et al., 2010
*Triticum aestivum* L.	Put foliar application	Improved antioxidant system for drought tolerance	Gupta et al., 2012
*Zea mays*	Put priming application	Improved cell division in apical meristem and enhanced seedling growth.	Hussain et al., 2013
*Trifolium repens* L.White clover	Spd priming	Improved the antioxidant defense system by regulating SOD, CAT, POD, and APX, and the ascorbate-glutathione cycle (ASC-GSH cycle) via inducing transcription level of antioxidant enzyme-related genes.	Li et al., 2014
*N. tabacum*	Put treatment	Promoted the photochemical capacity and reducing cellular dehydration.	Kotakis et al., 2014
*Valeriana officinalis* L.	PAs (Spd and Spm) application after transplanting	Triggered the antioxidant enzyme system and proline accumulation to reduce membrane damages and protect photosynthetic pigments for energy production.	Mustafavi et al., 2016
*Triticum aestivum* L.	PAs (Spd, Spm, Put) application for seed soaking	Improved endogenous indole-3-acetic acid (IAA), zeatin (Z)+zeatin riboside (ZR), abscisic acid (ABA), and gibberellin (GA) content in seeds, enhanced starch degradation and improved soluble sugars during seed germination.	Liu et al., 2016b
*Triticum aestivum* L.	Put, Spd, Spm (100 µM) seed priming for 10 h	Elevated the osmolytes accumulation and free polyamine contents through regulating polyamine biosynthetic genes.	Ebeed et al., 2017b
*Eleusine coracana* L. Gaertn.	Spd spray (0.2 mM) during 3 weeks at early flowering stage	Enhanced proline accumulation to relieve from drought stress.	Satish et al., 2018
*Rosa damascena*	PAs (Spd, Spm) foliar application	Regulated cellular water status, proline content, chlorophyll content, CAT and SOD activity, and stomatal conductance.	Hassan et al., 2018
*Lactuca sativa* L.	Put foliar application on seedlings	Regulated stomatal movement, and improved the ultrastructure of cell to reduce plasmolysis.	Zhu et al., 2019
*Vigna radiata* (L.) Wilczek	PAs (Put, Spd, Spm or combined application) priming treatment	Enhanced the level of total soluble sugar and protein, regulated water status and chlorophyll content, improved proline concentration and reduced MDA level.	Sadeghipour, 2019
*Triticum aestivum* L.	PAs (Put, Spm and combination of both) priming and foliar application	Improved chloroplast ultrastructure that improves the activity of Rubisco and photosynthetic pigments, increased CAT activity, reduced ROS and MDA level and protected the mesophyll cells.	Hassan et al., 2020

### Polyamines and salt stress

Salt stress is a complex environmental constraint that limits agricultural productivity (Ahmed et al., 2022). A higher salinity level alleviates the membrane integrity and enzyme activity and also impairs the functioning of photosynthetic machinery. Plants acclimatize to non-conducive environmental cues by accruing osmotic substances, including proline and PAs. The treatment with different types and levels of PAs such as exogenous Spd has been shown to lessen the impact of salinity conditions on different plants, where it lessens the damage via the accrual of proline and enzymatic antioxidants, besides regulating ion exchange (Kamiab et al., 2014; Lou et al., 2018; Zhang et al., 2018); several reports are shown in **Table 2**.

**Table 2:** Exogenously applied polyamines induce salinity stress tolerance in different plant species.

Polyamines	Ways of action	Studied plants	References
Put	It relieved the plant from salt stress by maintaining water and nutrition status.	Cucumber	Shu et al., 2012
Spd	Promoted γ-aminobutyric acid accumulation.	Soybean	Fang et al., 2020
Spd	May participate in the redox homeostasis, hence stability of chloroplasts against salt stress.	Rice	Jiang et al., 2020
Bound polyamines	Accelerated enzymatic antioxidant mechanisms and regulated TGase to enhance salinity tolerance.	Tomato	Zhong et al., 2020
Spd	Enhanced proline accretion and antioxidant enzymes efficiency for defense against salt stress	Alfalfa	Lou et al., 2018
Spd	Improved the antioxidant system and markedly enhanced Spd and Spm content.	Wheat	El-Sayed et al., 2018
Spd	Helped in the protection of photosynthetic machinery to maintain photosynthesis.	Tomato	Hu et al., 2016
Spd	Amended CAT activity and enhanced the photochemical quantum yield plants.	Pot marigold	Baniasadi et al., 2018
Spd	Effectively upregulated the transcription of Calvin cycle genes in order to improve and regulate the defense response.	Sweet sorghum	Sayed et al., 2019
Put	Reduced salt-induced photosynthetic perturbation of leaf through stomatal-aperture modifications.	Cucumber	Ma et al., 2020

Kasinathan and Wingler (2004) showed the effect of reduced arginine decarboxylase activity on salt tolerance and on polyamine formation during salt stress in *Arabidopsis thaliana*. Additionally, Liu et al. (2006) proved the importance of the arginine decarboxylase pathway in the stress response of apple callus. PAs generally showed strong salt tolerance and mutants in the synthesis of spermine and thermospermine (*acl5-1*, *spms-1*, and double *acl5-1*/*spms-1*) accumulated higher Na^+^ content and restricted growth than control plants (Alet et al., 2012). PA (Spm and Spd) application increased ROS metabolism and photosynthetic efficiency, which enhanced plant development and reduced the negative impact of salinity stress (De-Yun et al., 2015; Baniasadi et al., 2018). The free, acid-soluble, bound, and total Spm content in leaf was enhanced under salt stress (50, 100, and 150 mM NaCl) in *Helianthus* plants (Mutlu and Bozcuk, 2005). The enhancement of Spd and Spm, besides the reduction of Put content, resulted in the improved growth of sunflower seedlings (Takahashi et al., 2018). Several metabolic processes are positively influenced by exposure to Spm and Spd via regulating the expression level of genes related to a defense system (antioxidant and osmolyte biosynthesis-responsible genes), ion transport (Na^+^/H^+^ antiporter gene; *NHX1*), late embryogenesis abundant (*LEA*) and PA metabolism-related enzymes in rice seedlings (Paul and Roychoudhury, 2017). An earlier study characterized the polyamine oxidases (PAO) family into four genes (*CsPAO1*-*CsPAO4*), which are important in plant growth; meanwhile, *CsPAO3* is a candidate gene enhancing salt tolerance (Wu et al., 2022).

Previous reports demonstrated that exogenous PA applications can enhance salt tolerance, whereas Put improve the photosynthesis in cucumber plants by improving photochemical efficiency, consequently ameliorating the adverse effects of NaCl (Zhang et al., 2009). Likewise, PA treatment under salt stress significantly affected the accumulation of secondary metabolites involved in redox balance, particularly flavonoids and other phenolics (Buffagni et al., 2022). Exogenously supplied Put alleviated the Na^+^ accretion in salt-sensitive rice cultivar under salt stress, increased Put biosynthesis, and a higher level of conjugated PAs in stressed tissues counteracted salinity stress (Quinet et al., 2010). Moreover, Put activated the genes encoding amine oxidases and elevated the ethylene synthesis in salt-stressed plants (Quinet et al., 2010). Seed priming with Put is an efficient way to improve seed germination under salt stress, evidenced in chamomile and sweet marjoram plants grown under salinity stress (Ali et al., 2009).

Exogenous Spd application enhanced salt tolerance by improving the activity of enzymatic antioxidants and proline content in cucumber and ginseng seedlings (Duan et al., 2008; Parvin et al., 2014). Likewise, a shielding upshot of supplied Spd was noticed in two Kentucky bluegrass cultivars through higher antioxidant enzyme activity and reducing lipid peroxidation (Puyang et al., 2015). In chrysanthemum seedlings, Na^+^ uptake was reduced, besides osmotic and ionic balance, antioxidant quenching ability, membrane integrity, and photosynthetic efficiency (Zhang et al., 2016a). Seed soaking with Spd displayed a higher germinating rate and seedling development via reducing chlorophyll damage and enhancing anthocyanin and phenolic compounds in addition to alleviating H_2_O_2_ and Pro contents in rice (Chunthaburee et al., 2014). In tomato roots, the Spd pretreatment increased the conversion of free Put into free Spd and Spm and altered the metabolic status of PAs, hence reducing salinity stress (Hu et al., 2012). In bermudagrass, PAs induce various processes that could improve salt tolerance via mediating several processes such as energy and electron transportation, the antioxidant system, and the carbon fixation pathway (enhance osmolyte synthesis), which might apply for genetically engineered crops to enhance stress adaptation (Shi et al., 2013).

Additionally, Spd application reduced the superoxide production and lipid peroxidation and improved the ascorbate-glutathione cycle components, resulting in the reduction of damage in tomato plants with salinity-alkalinity exposure (Zhang et al., 2016b). In cucumber, a beneficial impact of Spd application on photosynthesis was associated with higher tolerance under salt stress (Sang et al., 2016); moreover, exogenously supplied Spd also enhanced salinity tolerance in zoysia grass through improving PA metabolism by increasing arginine decarboxylase (ADC), S-adenosylmethionine decarboxylase (SAMDC), and diamine oxidase (DAO) activities (Li et al., 2017). In sweet sorghum, Spd application increases the photosynthetic efficacy by inducing the transcript level and activity of CO_2_-assimilating enzymes [ribulose 1,5-bisphosphate carboxylase/oxygenase (Rubisco) and aldolase] in plants exposed to saline conditions (Sayed et al., 2019).

Exogenously supplied Spd enhanced plant growth and biomass by stimulating the antioxidative system in response to saline conditions in soybean (Fang et al., 2020). Furthermore, in rice, Spd exposure prohibited the stress damage to chloroplasts’ ultrastructure and photosynthetic apparatus by inducing an antioxidant defense system, indicative of Spd participation in redox homeostasis (Jiang et al., 2020). Spd application boosted the accretion of endogenous GA by stimulating the responsible gene level related to GA production and elevating gibberellin oxidase functioning, which enhanced the expression of the GT-3b transcription factor. Furthermore, GA facilitated the Spd-induced salinity tolerance in cucumber plants (Wang et al., 2020). Exogenous application of Spd resulted in salt tolerance through inducing *ATG* (autophagy-related genes); moreover, it activated RBOH that enhances H_2_O_2_ generation, which activates autophagy, thereby improving salt stress tolerance (Zhang et al., 2020). In addition, exogenous Spd significantly reduced the adverse effects of salt stress in tomato seedlings via inducing the H_2_O_2_-mediated signaling pathway that is involved in increasing RBOH1 expression level and salt stress-responsive genes *SlMYB102*, *SlHKT1*, *SlWRKY1*, and *SlDREB2*, thus enhancing detoxification by improving antioxidative activity and osmotic substances (Raziq et al., 2022).

**Figure 4:**
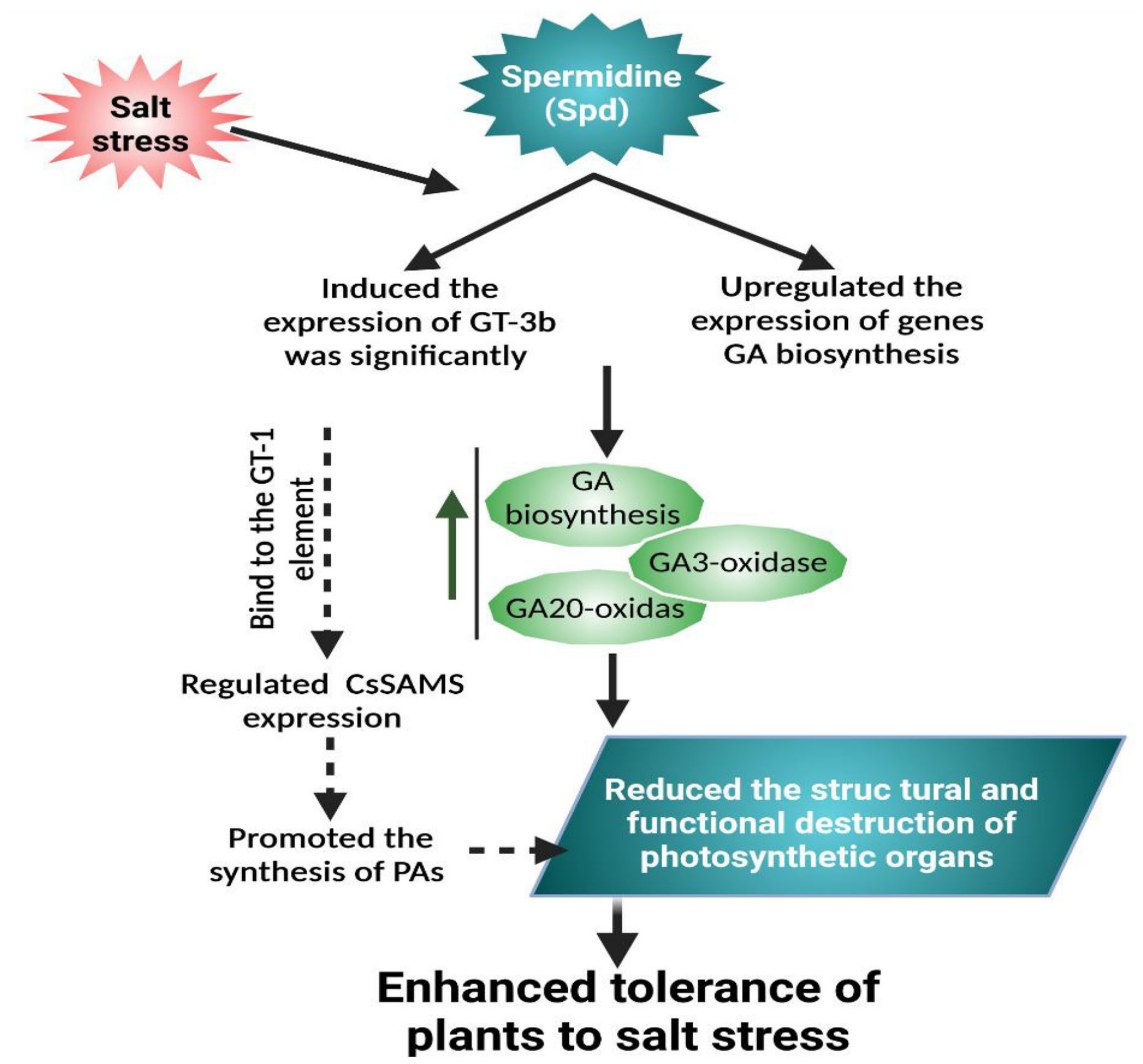
Exogenously applied Spd can improve salt tolerance. Spd-enhanced GT-3b expression level that is markedly related to endogenous GA content. Exogenous Spd significantly increased the content of endogenous GA3 under salt stress, upregulated the expression of genes related to GA biosynthesis, and enhanced the activity of GA3-oxidase and GA20-oxidase. GT-3b can bind to the GT-1 element in the promoter of the S-adenosylmethionine synthase gene (CsSAMS) to regulate CsSAMS expression in cucumber, which can promote the PA synthesis. The PA and phytohormones interplay alleviated the disruption of photosynthetic organs and promoted stress tolerance (Wang et al., 2020).

Spermidine as a plausible candidate for salt resistance in cucumber seedlings is illustrated in **Figure 4**. Under salt stress, exogenously applied Put reduced stress by improving protein synthesis at transcriptional and translational levels; moreover, Put treatment results in higher endogenous PA content, especially Spd and Spm, resulting in elevated fatty acid mobilization that is attributed to stabilizing photosynthetic machinery (Shu et al., 2015). Furthermore, omics studies exposed that Put has the ability to restore root development by improving the transcript level of proteins encoding genes related to plant defensive responses and carbohydrate and amino acid metabolism, which leads to stress tolerance and energy production in cucumber seedlings under exposure to saline conditions (Yuan et al., 2016). In cucumber, exogenously applied PAs (especially Put) enhanced the endogenous PA content through ADC pathways by the induction of *AGPase* and *BAM1* gene expression, which resulted in the regulation of AGPase and β-amylase enzyme activities that are involved in starch metabolism and decreased starch overaccumulation in leaf, thereby protecting the photosynthetic pigments and improving salt stress tolerance (Shen et al., 2019).

**Figure 5:**
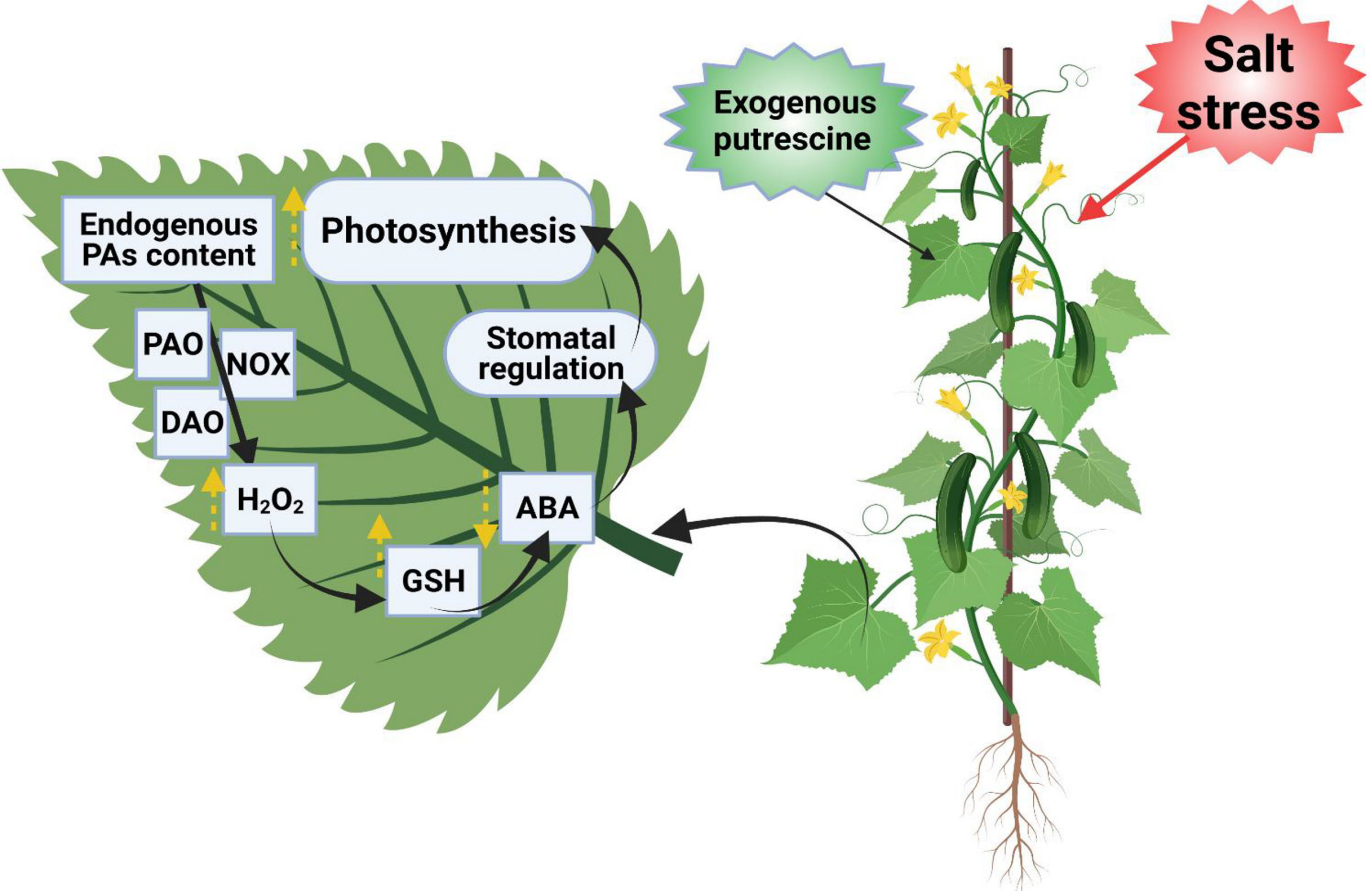
The model diagram of exogenous Put effects on the photosynthesis of cucumber leaves under salt stress. It regulates the stomatal opening and closing, where: Put-putrescine, PAs-polyamines, DAO-diamine oxidase, PAO-polyamine oxidase, NOX-nicotinamide adenine dinucleotide phosphate oxidase, H_2_O_2_-hydrogen peroxide, GSH-reduced glutathione, ABA-abscisic acid (Ma et al., 2020).

Under salt stress, Spm levels were enhanced at the expense of Put and Spd, suggesting a sophisticated mechanism leading to the biosynthesis of polycations as stress sensors; additionally, the equilibrium between high and low polycationic forms was weakened in the salt-sensitive genotype TN6.18, displaying a strong correlation with its sensitive phenotype (Antoniou et al., 2021). Put application regulates stomatal changes via inducing H_2_O_2_ signaling that mediates PA degradation by diamine oxidase (DAO), which enhances GSH level and hampers ABA accumulation under salt-stress conditions (Ma et al., 2022). The model diagram of exogenously applied Put indicates the improved photosynthesis of cucumber leaves by regulating stomatal movement under salt stress (**Figure 5**).

### PAs and oxidative stress

Polyamines play a complex role in plant oxidative stress (Minocha et al., 2014). PAs can accelerate the functioning of the enzymatic antioxidant defensive system, which is associated with efficient regulation of oxidative stress in plants on exposure to environmental adversities (Wang et al., 2020; Hasan et al., 2021a). Spm and Put treatment in maize leaves revealed higher tolerance against oxidative stress induced by paraquat (Durmuş and Kadioğlu, 2005). Exogenously applied Spd enhanced Spd and Spm while alleviating the Put level in cucumber roots under hypoxia stress, which was attributed to efficient enzymatic antioxidant activity, higher ROS detoxifying ability, and lower MDA levels, eventually improving stress resistance (Wu et al., 2018). Exogenous PAs (1mM), such as with Spm application, ameliorated the effects of Cd^2+^ and Cu^2+^ on lipid peroxidation (Tajti et al., 2018). Likewise, GR activity was significantly restored by Spm or Spd treatments, whereas SOD activity under Cu^2+^ application was re-established by Spm treatment.

Nevertheless, PAs are a source of ROS due to their catabolism that generates the strong oxidizers H_2_O_2_ and acrolein, which might be responsible for cellular breakdown under stress (Minocha et al., 2014). Meanwhile, H_2_O_2_ is also a signaling molecule involved in the stress signal transduction chain and stimulates an antioxidant defense response (Groppa and Benavides, 2008). Thus, it seems that PAs are regulators of redox homeostasis that play a dual role in plant oxidative stress (Groppa et al., 2001; Saha et al., 2015).

### PAs and metal stress

Plants are sessile organisms and are exposed to various environmental conditions and affected by stresses such as heavy metal stress (Tajti et al., 2018; Naz et al., 2021), besides various diseases and pests (Khajuria and Ohri, 2018). Put exhibited multifaceted biochemical properties and its important functions under K^+^ deficiency have been documented, such as cation balance (K^+^ and Ca^2+^), antioxidant activities, ROS-mediated signaling, osmolyte accumulation, and pH regulation; moreover, Put and its catabolites regulate ionic balance, and mitochondria and chloroplast bioenergetics under K^+^ stress (Cui et al., 2020). Spd application protected the thylakoid membrane, induced antioxidant enzyme activities, enhanced the photosystem II (PSII) efficiency, depressed the expression of Chl catabolism-related genes, and dramatically reduced the generation of free radicals (O^2·-^), hydrogen peroxide (H_2_O_2_), and MDA in chloroplasts, thus protecting against chlorophyll (Chl) losses under aluminum (Al^3+^) stress (Jiang et al., 2021).

Exogenous application (seed soaking or foliar application) of PAs substantially enhanced plant growth and yield under Cd^2+^ and Pb^2+^ stress via improving defense mechanisms and reducing power capacity in wheat plants (Taie et al., 2019). In another study, PAs addressed Cd^2+^-induced destruction by improving tolerance through integrity of cell structure via modulating nutrients and regulating the ammonium/nitrate ratio in *Inula crithmoides* (Ghabriche et al., 2017). Under stress, PAs involved in stress signaling modulate ionic balance and regulate ion transportation through ion channels (Podlešáková et al., 2019). Furthermore, PAs are one of the important components involved in proper growth and physiological processes as well as possess a vital contribution to abiotic stress tolerance (Mirza Hasanuzzaman et al., 2014).

Higher levels of endogenous Spd and Spm during Cd^2+^ stress ameliorate the adverse effect in wheat that indicates their protective role against metal/metalloid(s) stress (Howladar et al., 2018). Under metal stress, the plant responds by enhancing endogenous PAs, whereas exogenous PA application facilitates better metal tolerance for the plant (Nahar et al., 2016). Additionally, mung bean, on Cd^2+^ stress, responds through PA-induced defense, where Put (0.2 mM) against 1.5 mM Cd exposure results in a lower biological accumulation coefficient (BAC), translocation factor (TF), and biological concentration factor (BCF), resulting in relief from Cd^2+^ stress in root and shoot tissues (Nahar et al., 2016). It was found that PAs induced lower metal/metalloid(s) accumulation, and also assisted in metal translocation, besides the synthesis of phytochelatins (successfully binds metals) that are the reason behind lower toxicity under metal stress (Nahar et al., 2016). Phytochelatin biosynthesis takes place through GSH that works as a substrate to form phytochelatins, which bind with metal ions and are transported in the vacuole for the protection of cell from toxic ions (Nahar et al., 2016).

Previous studies reported that exogenously applied PAs modulate the antioxidant defense system to scavenge excessive ROS (Paul et al., 2018). Under Cd stress, the exogenous application of Put detoxify the free radicals (ROS) by enhancing enzymatic antioxidant enzyme efficiency (SOD, CAT, APX, MDHAR, DHAR, GR, GST, and GPX) and nonenzymatic antioxidants (AsA and GSH) (Nahar et al., 2016). The exogenously treated *Boehmeria nivea* L. plant with Spd showed tolerance against Cd^2+^ stress (Gong et al., 2016): besides, Spm or Spd priming in wheat facilitated the seedlings to combat Cd^2+^ stress (Rady and Hemida, 2015). Seed soaking or foliar application of PAs significantly enhanced plant growth and yield under Cd^2+^ and Pb^2+^ stress by improving defense processes in wheat (Taie et al., 2019). The toxic effect of Cd^2+^ and Cu^2+^ altered the membrane fluidity in leaf and root during early seedling growth; however, pre-treatment with PAs ameliorated the toxic effects in plants (Benavides et al., 2018). Additionally, PA application enhanced the level of free, soluble, and insoluble conjugated PAs that facilitate the tolerance under Cr^2+^ toxicity in Kinnow mandarin (Shahid et al., 2018). The higher level of PAs might be attributed to the higher activity of PAs’ anabolism-related enzymes (ADC, ODC, SAMDC, and SPDS) along with lower catabolic enzymes (polyamine oxidase: PAO and DAO) on metal stress (Shahid et al., 2018). The higher level of GSH is related to higher PA level, and GSH itself participates in toxic metal chelation, leading to metal detoxification (Howladar et al., 2018). Numerous studies evidenced the PA-induced GSH level as a tolerance mechanism for metal/metalloid stress (Rady and Hemida, 2015; Nahar et al., 2016; Howladar et al., 2018). On metal stress, Put application provides the osmotic protection through elevating Pro levels, water status, and chlorophyll synthesis (Nahar et al., 2016). The higher Pro level in plants might be due to increasing activities related to Pro synthesis for ameliorating metal stress (Amist and Singh, 2017). Several studies documented that PAs improved the photosynthetic efficiency and protected the photosynthetic apparatus under stress conditions (Sheng, 2012; Balal et al., 2017; Kumar et al., 2017).

Seed treatment with PAs led to improved functioning in wheat under Cd^2+^ stress; moreover, 0.25, 0.50, and 1.0 mM of Spm, Spd, and Put, respectively, were used to enhance plant growth on exposure to 2.0 mM Cd^2+^. In conclusion, 1.0 mM Put application was the best one for stress response by improving RWC, membrane integrity, photosynthetic pigment synthesis, nutrient level, and by providing osmoprotection (Rady et al., 2016). Moreover, PAs (Put, Spd, and Spm) reduced the toxic effects of Cd^2+^ in *Inula crithmoides* by maintaining cellular structure via modulating nutrient uptake and ammonium/nitrate ratio (Ghabriche et al., 2017).

Metal/metalloid(s) stress also induced oxidative stress by excessive ROS production, ionic imbalance, and ROS-induced disruption and disturbance in lipid, nucleic acid, and protein (Nahar et al., 2016). Nonetheless, plants exhibited the antioxidative defense response against oxidative stress by activating the antioxidant system; however, the capacity may reduce due to stress toxicity (Nahar et al., 2016). Under that situation, PAs can actively take part in ameliorating oxidative stress by reducing ROS generation (Baniasadi et al., 2018; Hassan et al., 2018). Occasionally, H_2_O_2_ can be generated by the activity of DAO and PAO enzymes to degrade PAs that cause the activation of the antioxidative defensive system. Additionally, PAs exhibited both anionic and cationic binding sites, which assist in radical quenching and improving antioxidant properties, eventually reducing lipid peroxidation and oxidative reactions (Groppa and Benavides, 2008). Polyamines are involved in the binding of anions (phospholipid membranes and nucleic acids) in the cell, particularly prone to oxidations, while the cations efficiently prevent the generation of site-specific ROS, such free radicals (LØVaas, 1996). Previous studies reported that PAs might protect the membranes from oxidative damage through a complex of PAs, phospholipids, and Fe^2+^, which can prevent Fe^2+^ auto-oxidation (Velikova et al., 2000). Likewise, Al^3+^ causes stress, whereas Put treatment decreases the activity of H_2_O_2-_producing enzymes known as the CW-PAO (cell wall-PAO) and plasma membrane NADPH oxidase (Yu et al., 2018). The conjugate molecules of PAs play an important role for scavenging harmful radicals, and phenylpropanoid-PA conjugates can react with reactive nitrogen species (RNS) and ROS, and improve the antioxidant enzyme activity for metal detoxification (Shahid et al., 2018). Numerous studies revealed the protective role of exogenous PA application under stress conditions by activating the antioxidant defense system for ROS quenching (Nahar et al., 2016; Sánchez-Rodríguez et al., 2016a; Hassan et al., 2018). Besides, Nahar et al. (2016) found that PAs detoxified the toxic methylglyoxal (MG) by improving the glyoxalase system through the upregulation of glyoxalase I (Gly I) and glyoxalase II (Gly II) enzymes, indicating the indirect role for reducing oxidative stress. Taken together, The lowering of oxidative stress either directly or indirectly in plants is attributed to PAs.

**Figure 6:**
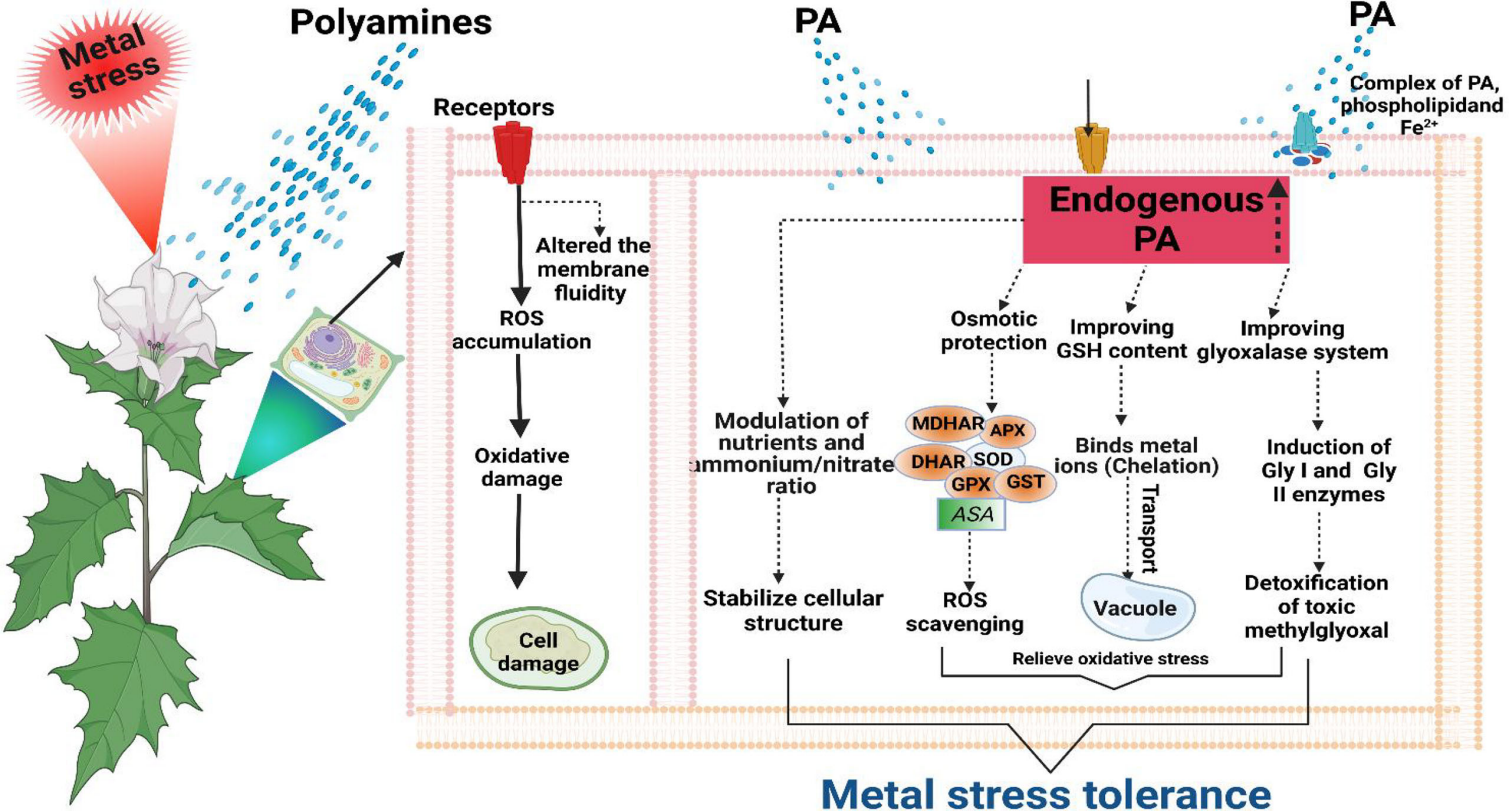
Metal stress tolerance with the exogenous application of PAs. Protecting the membrane structure by forming complexes with phospholipid and Fe^2+^. PAs improve the glyoxalase system through inducing glyoxalase I (Gly I) and glyoxalase II (Gly II) enzymes that detoxify the toxic methylglyoxal. Enhances the glutathione (GSH) level, which involves metal chelation and transportation into vacuole for detoxification. Improves the activity of enzymes (SOD, superoxide dismutase; CAT, catalase; APX, ascorbate peroxidase; MDHAR, monodehydroascorbate reductase; DHAR, dehydroascorbate reductase; GR, glutathione reductase; GST, glutathione S-transferase; GPX, glutathione peroxidase) and nonenzymatic antioxidants (ascorbate, AsA). Hence, reduced oxidative stress directly and indirectly. Maintaining the cellular structure through modulation of nutrition and ammonium/nitrate ratio.

Polyamines contributed to maintaining redox homeostasis by elevating the AsA and GSH levels under metal toxicity in plants such as Spd in *R. sativus* upon Cr^2+^ exposure (Choudhary et al., 2012). Under Cd stress, the free PA pool was enhanced besides the higher AsA and GSH pool involved in ROS scavenging (Yang et al., 2010). In crux, the above discussion revealed that PAs modulate the antioxidant system under metal/metalloid(s) toxicity by inhibiting ROS-induced oxidative damages (**Figure 6**).

## Conclusion and future remarks

Climate change is leading to stressful environmental cues, which is increasingly wearing down crop production and yield. Accordingly, the major significance of plant research related to resistance mechanisms is dramatically progressing, providing solutions with the generation of stress-tolerant cultivars. To gain insight, polyamines tying in with a defensive mechanism to combat stress stimuli is an emerging theme in the field of research; however, the understanding of this interplay is complicated. This review aims to present recent indications regarding abundant PA (putrescine, spermidine, and spermine) contributions in metabolic and physiological processes to protect plants during climatic adversities. PAs are known as protective molecules, besides being important participants in a complex signaling system and having a vital role in tolerance mechanisms, depending on their type and concentration under stress conditions. PAs are involved in several physiological and metabolic processes, including photosynthetic pigment defense, antioxidant systems, hormonal interplay, and ionic homeostasis, which ultimately ameliorate the negative effects of non-optimal conditions on plants. This paper identifies PA hubs that improve the diverse stress acclimation mechanisms that consequently enhance crop growth, development, and productivity under adverse conditions. There are several unresolved questions regarding the roles of PAs in regulating plant growth and development. To conclude, a huge gap of knowledge regarding biosynthetic and catabolic processes related to abiotic stress acclimation in plants is still developing, and it is obvious that transcriptional, translational, and post-transcriptional approaches should now be attempted for understanding and manipulation. Additional efforts are needed to be invested to both complement and guide breeding and genetic manipulation programs by having a comprehensive understanding of the clear participation of PAs to enhance stress tolerance. Likewise, it is desirable that future work exploit further synergies to be gained about the metabolic relationship between PAs and hormonal interplays during plant development, especially under stress conditions. With the advancement of molecular biology techniques, research is now focusing on events at the molecular level. Illuminating the regulation mechanism of PAs at the molecular level should be an important future research direction besides exogenous PA application for rational improvement in crop production under stress conditions. The recognition of PA-regulated downstream targets, such as PA-responsive factors and transacting proteins, and the discovery of the integration between PAs and stress-responsive elements can open up new themes in the field of research to elaborate the important roles of individual PAs at the transcriptional, translational, and molecular levels.

## Author contributions

Conceptualization, JS; writing–original draft preparation, JS, MB and GH; writing–review and editing, KH, FI, RA, MH, HAN, WW, QU, XL, MA, MUH and AES. All authors contributed to the article and approved the submitted version.

## Funding

This research was funded by the National Key Research and development Program of China (2016YFD0300208) and the National Natural Science Foundation of China (41661070); Guangxi Key R&D program (Guike AB19245040) and Guangxi Key R&D program (Guike AB19245039); Guangxi Key Laboratory of Water Engineering Materials and Structures fund program (GXHRI-WZMS-2020-03); Key disciplines (construction) of ecology in the 13th Five-Year Plan of Jiangxi Agricultural University.

## Conflict of interest

The authors declare that the research was conducted in the absence of any commercial or financial relationships that could be construed as a potential conflict of interest.

## Publisher’s note

All claims expressed in this article are solely those of the authors and do not necessarily represent those of their affiliated organizations, or those of the publisher, the editors and the reviewers. Any product that may be evaluated in this article, or claim that may be made by its manufacturer, is not guaranteed or endorsed by the publisher.
